# Attitudes of the general population and mental health practitioners towards blended therapy in Austria

**DOI:** 10.1007/s00508-024-02391-9

**Published:** 2024-07-22

**Authors:** Gloria Mittmann, Verena Steiner-Hofbauer, Beate Schrank

**Affiliations:** 1 Research Centre Transitional Psychiatry, Karl Landsteiner University of Health Sciences, Dr. Karl-Dorrek-Straße 30, 3500 Krems, Austria; 2 Department of Psychiatry and Psychotherapeutic Medicine, University Hospital Tulln, Tulln, Austria

**Keywords:** Psychiatry, Blended care, Blended counselling, M‑health, E‑health

## Abstract

**Introduction:**

Mental health problems are steadily increasing worldwide. In Austria, the overall supply of mental health services is low, especially in rural areas. Mobile technology and a blended care approach have the potential to overcome problems with service provision. The aim of this study was to map the attitudes of practitioners and people living in Austria towards blended therapy.

**Method:**

Two individual online questionnaires (including the Unified Theory of Acceptance and Use of Technology, advantages and disadvantages, useful features) were distributed to practitioners and the general population in Austria.

**Results:**

The questionnaires were answered by 152 members of the general public and 129 practitioners. The general population and practitioners seem to be cautious, but slightly positive about blended therapy. Previous experience of practitioners with blended therapy was low. Practitioners are most worried about the therapeutic process and their work-life balance, while the general population is worried about being overwhelmed by the concept, mainly due to the time investment. Tracking, recording and reminding functions (e.g., for mood, homework) were seen as especially valuable features and accessibility was deemed the biggest advantage by both samples.

**Conclusion:**

Practitioners’ attitudes are important for implementation of blended therapy. More awareness might help against the cautiousness as well as implementing digital health applications in Austrian health policies.

**Supplementary Information:**

The online version of this article (10.1007/s00508-024-02391-9) contains supplementary material, which is available to authorized users.

## Introduction

Mental health problems are steadily increasing worldwide. For example, the prevalence of both depression and anxiety showed rising trends during the last years [1, 2]. Young adults are especially vulnerable after the coronavirus disease 2019 (COVID-19) had an additional detrimental effect on mental health in adults [3]. Mental illness has become a primary factor contributing to the burden of disease among young individuals in high-income countries [4]. In Austria, the supply of mental health services, institutions and specialists is low compared to the need for such services in the population [5, 6].

Technological approaches have the potential to overcome problems with service provision [7], for example by reaching patients in more rural areas. One option for using technology is mhealth apps—mobile apps that “are software programs on mobile devices that process health-related data on or for their users” [8].

In general, mhealth apps seem to be beneficial in a mental health context, especially concerning depression and anxiety [9, 10]. For example, Rathbone et al. [11] found in their systematic review that apps based on cognitive behavioral therapy (CBT) had a positive impact on several mental health issues such as symptom severity and Firth et al. [12] found in their review with 18 randomized controlled trials (RCT) that mhealth apps significantly reduced depression symptoms compared to control groups. There is also some evidence that digital interventions and mhealth apps can be effective in suicide prevention [13].

Most of these apps which apply to a therapeutic context are more likely to be used as an alternative to traditional face-to-face therapy rather than an addition to in-person therapy. In a review about downloadable Android and iOS apps for psychosocial well-being, less than 2% (19/1009) of the apps were designed as a supplement to in-person psychotherapy [14].

Using supplementary apps in an ongoing therapeutic process is called blended therapy, blended care or blended counselling. Blended therapy means combining elements of both face-to-face and internet interventions [15].

A decisive factor for therapeutic success is the therapeutic relationship [16]. A fear around mhealth is the lack of therapeutic relationship due to the online character of the therapy, including the loss of therapeutic alliance. Contrary to that belief, ehealth therapy was found to be equally as good as traditional therapy [17]. Pihlaja et al. [18] found in their systematic review about etherapy for depression and anxiety that the therapeutic alliance is not only as good as in face-to-face therapy but might even exceed it.

Research found that most mhealth apps are usually found via social media, personal searches and private recommendations and not due to recommendation by professionals [19]. Furthermore, acceptance of mhealth apps among healthcare providers is still moderate to low [20]. This is due to various barriers experienced by health professionals, such as technical, social and organizational problems. Most prominently, fears around workload and training are factors impacting adoption [21]. Yet, staff play a central role for the uptake of mhealth apps [22] and engagement from both sides is crucial for continued use [23]. Other research around potential barriers found that technical problems are often considered a limitation [22, 24, 25]. Yet, attitudes towards mhealth apps might depend on the country and even the region people live in.

In 2016, Schuster et al. [26] explored differences of advantages and disadvantages of online and blended therapy with Austrian psychotherapists. They found that most therapists preferred blended therapy over online therapy. The current study expanded their study in several ways: First, it included an open question format to gather more qualitative data on practitioners’ attitudes. Second, it included a broader spectrum of occupations, including psychiatrists and clinical psychologists. Third, it re-evaluated the topic after the COVID-19 pandemic, where on the one hand mental illnesses have become more prevalent and on the other hand digital technologies have gained rapid adoption due to their necessity during lockdowns. While Schuster et al. [26] found an increased perception of disadvantages of web-based interventions, this might have changed in the population of practitioners during the last years. Lastly, to our knowledge, no one has mapped attitudes towards mhealth apps in a general population of adults in Austria.

Blended therapy is not widely common in Austria. Yet, due to its rural characteristics, mhealth apps especially in a blended approach could potentially be an advantageous addition to mental health services to be able to reach people. This exploratory study aimed to map the attitudes of Austrian adults with and without mental illnesses and practitioners of the mental health sector towards blended therapy and the use of an mhealth app.

## Methods

### Research question

The research question was: what are the attitudes of practitioners and people living in Austria towards a blended therapy approach using an mhealth app? Due to the exploratory nature of the study, no hypotheses were formed beforehand. For handling missing data, listwise exclusion was chosen.

### Study procedure

Two individual online questionnaires were distributed to practitioners and the general population in Austria. Each questionnaire took about 5 minutes to complete. Participants indicated their consent at the beginning of the questionnaire. Our convenience sample was recruited via social media, sharing with our network and, in the case of practitioners, actively approaching hospitals and institutions with psychological or psychiatric care.

### Material

The questionnaire started with sociodemographic data, asking for sex, age, place of residence and potential diagnosis of mental illness. We then used a version of the unified theory of acceptance and use of technology (UTAUT) adapted to a clinical setting (with excellent model fit; RMSEA = 0.035, SRMR = 0.029) [27, 28]. This scale has five subscales with a Likert answer format between 1 (not at all) to 5 (completely), covering performance expectancy (how blended therapy influences or benefit the therapy, effectiveness of treatment, improvement of the therapeutic process, therapy outcomes or productivity of the therapist), effort expectancy (how much additional effort is needed, handling, usability, workload and integration of blended therapy), facilitating conditions (expected technical or organizational support while using the technology, financial and technical conditions), social influence (influence of advice and recommendations to use or not to use blended therapy) and internet anxiety (anxiety towards the internet in general). The questionnaire was originally used to examine attitudes about blended therapy in clinical experts. For the general population, we adopted the subscales of performance expectancy, effort expectancy, facilitating conditions and internet anxiety. The subscale social influence was only handed to the sample of practitioners as the items relate to colleagues from the mental health sector. As a benchmark, the neutral answer on the Likert scale (3) was used to distinguish between agreeing and not agreeing. We then asked for both advantages and disadvantages of blended therapy, with options extracted from the relevant literature as well as open questions for personal opinions. Finally, we asked for features that participants would like to see implemented in a mhealth app, including space for participants’ own suggestions. The full questionnaire for both practitioners and the general population can be found in the supplementary material.

### Analysis

Due to the exploratory nature of the study, the data were analysed descriptively. All data are reported in means and standard deviations or percentages of participants.

## Results

### Sample

The general population questionnaire was answered by 152 participants; of these, 105 were female, 42 male, 3 diverse and 2 persons did not answer. Mean age was 41.02 years (SD ± 12.94 years), 23% were between 18 and 30 years old, 52% between 31 and 50 years and 25% between 51 and 75 years. The questionnaire for practitioners was answered by 129 participants; of these participants 102 were female, 16 male, 1 diverse and 10 persons did not answer. Mean age was 42.21 (SD ± 11.01), 17.6% were between 24 and 30 years old, 54.7% were between 31 and 50 years old and 27.7% were between 52 and 72 years old. Only 16.4% had experience with blended therapy.

Distribution of the sample between federal states, diagnoses of general population and work attributes of practitioners can be found in Table 1. Due to anonymity reasons, answers to the question of federal states were not mandatory for practitioners, which led to 86% missing data for that sample and question.

**Table 1:** Sample description for the general population and practitioners

		% of general population (*n* = 152)	% of practitioners (*n* = 129)
Federal states	Vienna	30.6	1.6
	Lower Austria	28.7	5.4
	Styria	23.6	1.6
	Tyrol	3.8	3.9
	Salzburg	2.5	n/a
	Burgenland	1.9	n/a
	Upper Austria	1.9	n/a
	Carinthia	1.9	n/a
	Voralberg	0.6	1.6
Diagnosis	None	52.6	–
	Depression	18.4	–
	Anxiety	8.6	–
	Other (e.g., depression together with comorbidities, bipolar disorder, OCD, borderline)	20.4	–
	Currently in therapy	34.4	–
Profession	Medical doctors	–	72.4
	Psychotherapists	–	7.9
	Psychologists	–	3.3
	(social) Pedagogue	–	2.6
	Others	–	13.8
Workplace	Hospital or clinic	–	85.7
	Private practice	–	12.4
	Practice with social insurance contract	–	0.8
Area of work	Children or adolescents	–	89.9
	Adults	–	10.1

*n/a* no answers, *–* not part of the questionnaire for this sample

### Unified theory of acceptance and use of technology

The overall average acceptance was M(mean) = 3.36 for practitioners and M = 3.75 for the general population (scale ranging from 1 to 5). The results of the UTAUT showed that internet anxiety was very low for both general population and practitioners (4.4 out of 5; the higher the rating, the lower the internet anxiety). In the general population, all other subscales were rated between undecided and slightly positive. For practitioners, facilitating conditions were rated more negatively compared to the other scales, while other subscales also ranged between undecided and slightly positive (see Table 2 for full results). Both practitioners with experience in blended therapy and people with a mental health problem indicated higher acceptance (Tables 2 and 3).

**Table 2:** Means of full sample of practitioners and practitioners with and without experience with blended therapy (BT) of the UTAUT subscales. Standard deviations in parentheses

UTAUT subscale	Practitioners *with* BT experience (*N* = 16)	Practitioners *without* BT experience (*N* = 75)	Full sample of practitioners (*N* = 91)
Performance expectancy^1^	3.8 (0.7)	3.4 (1.6)	3.5 (1.5)
Effort expectancy^2^	3.5 (0.8)	3.3 (0.7)	3.3 (0.7)
Facilitating conditions^3^	3.2 (0.6)	2.6 (0.7)	2.9 (0.7)
Social influence^4^	3.2 (0.7)	3.1 (0.6)	3.1 (0.6)
Internet anxiety^5^	4.6 (0.5)	4.4 (0.7)	4.4 (0.7)

Scale ranging from 1 not at all to 5 completely; higher values mean higher acceptance

^*1*^ Questions regarding effectiveness of treatment, improvement of the therapeutic process, therapy outcomes or productivity of the therapist, ^*2*^ Questions regarding handling, usability, workload and integration of BT, ^*3*^ Questions regarding financial and technical conditions, ^*4*^ Questions regarding the influence of advice and recommendations to use or not to use BT, ^*5*^ Questions regarding anxiety towards the internet in general

### Advantages and disadvantages of a blended therapy app

#### Advantages and benefits of a blended therapy app

The general population deems accessibility, reminder functions and consolidation of the therapeutic content the most beneficial. Practitioners’ highest rankings were for accessibility and tracking functions (such as mood monitoring). See Table 4 for all rankings.

**Table 3:** Means of the full sample of the general population (GP) and GP with and without psychological diagnosis of the UTAUT subscales. Standard deviations in parentheses

UTAUT subscale	GP *with* diagnosis (*N* = 48)	GP *without *diagnosis (*N* = 58)	Full sample of the general population (*N* = 106)
Performance expectancy^1^	3.5 (0.9)	3.3 (0.8)	3.4 (0.8)
Effort expectancy^2^	3.8 (0.7)	3.6 (0.7)	3.7 (0.7)
Facilitating conditions^3^	3.7 (0.7)	3.4 (0.6)	3.5 (0.6)
Social influence	–	–	–
Internet anxiety^4^	4.3 (0.9)	4.5 (0.8)	4.4 (0.9)

Scale ranging from 1 not at all to 5 completely, higher values mean higher acceptance

^*1*^ Questions regarding effectiveness of treatment, improvement of the therapeutic process, therapy outcomes or productivity of the therapist, ^*2*^ Questions regarding handling, usability, workload and integration of BT, ^*3*^ Questions regarding financial and technical conditions, ^*4*^ Questions regarding anxiety towards the internet in general

*–* not part of the questionnaire for this sample

Additionally, in the open question format, the general population mentioned the following advantages (exemplary): 24/7 availability, reduction of organizational effort, reduction of time effort, reduction of CO_2_ emissions, while practitioners suggested attractiveness to the patients, attractiveness to young patients and self-reflection between sessions as potential advantages.

#### Fears and uncertainties around a blended therapy app

For the general population, the highest rated fear or uncertainty was fear of being overwhelmed by the blended therapy app, followed by uncertainties about time investment. Practitioners were most uncertain about the therapeutic process, their work-life balance (through having to be available in the app) and the therapeutic relationship. See Table 5 for full ratings.

**Table 4:** Advantages/benefits of the use of a blended therapy app (scale: 1 very beneficial, 5 not at all beneficial)

	General population (*N* = 126) Mean (SD)	Practitioners (*N* = 90) Mean (SD)
Accessibility (rural areas)	1.58 (0.9)	1.90 (0.9)
Reminder function (medication, appointments,...)	1.71 (1.1)	–
Consolidation of therapeutic content	1.95 (1.0)	–
Tracking function (mood, habits, exercise,...)	2.13 (1.1)	1.96 (0.9)
Monitoring of assignments/homework	2.13 (1.1)	–
Educational videos	2.21 (1.2)	–
Deepening of the relationship between patient/therapist	2.93 (1.3)	3.12 (1.4)
Continuous engagement of patients (between sessions)	–	2.00 (0.9)
Deepening of the therapeutic process	–	2.14 (1.1)

*–* not part of the questionnaire for this sample

Additional suggestions for fears and uncertainties from patients (exemplary) included insufficient technical skills, insufficient technical equipment, and fear of mandatory use. Additional suggestions for fears and uncertainties from practitioners included insufficient technical equipment, fear of mandatory use, billability and lack of adherence.

#### Most useful features of a blended therapy app

Results show that 62.0% of the general population rated recording assignments or homework as most useful in a blended therapy app, while 70.4% of practitioners rated emergency contacts as the most useful feature. For both the general population and practitioners the fewest ratings were found for chat/online forum with other patients. For full ratings, see Table 6.

**Table 5:** Fears or uncertainties contracepting the use of a blended therapy app (scale: 1 very hindering, 5 not at all hindering)

	General population (*N* = 131) Mean (SD)	Practitioners (*N* = 90) Mean (SD)
Overloaded/Overwhelmed	3.23 (2.0)	2.76 (1.2)
Time investment	3.18 (2.3)	2.58 (1.1)
Money/Costs	2.87 (2.3)	–
Therapeutic relationship	2.84 (1.9)	3.30 (1.4)
Online contract not as good as offline contact	2.25 (1.8)	–
Data security	2.18 (1.9)	2.02 (0.9)
Billability	–	2.61 (1.2)
Depersonalisation	–	3.06 (1.3)
Work Life Balance	–	3.38 (1.5)
Therapeutic process	–	3.41 (1.5)

*–* not part of the questionnaire for this sample

**Table 6:** Features: most useful features of a blended therapy app

	General population (*N* = 137) %	Practitioners (*N* = 108) %
Recording assignments/homework	62.0	65.7
Recording and tracking of goals	58.4	61.1
Emergency contacts	58.4	70.4
Mood tracking	57.7	62.0
Habit tracking (exercising, meals, …)	54.7	67.6
Chat/video call with therapist	54.0	40.7
Recording of therapeutic content	51.8	50.9
Chat/online forum with other patients	23.4	27.8

In an open question format, participants could indicate other features they would like to see in a blended therapy app. The most frequently mentioned suggestions by the general population were a diary function for feelings and thoughts, cheering or encouraging messages or pictures, medication reminder/tracker, relaxation techniques and literature recommendations. For practitioners, the most frequently mentioned suggestions were motivation, reminder and tracking for relaxation techniques/exercises and emotion regulation/mindfulness/breathing exercises, cheering or encouraging messages, literature recommendations, diary function for feelings and thoughts and to do lists and weekly planning.

## Discussion

In this exploratory study, we investigated attitudes of both the general population and practitioners in Austria towards the use of a blended therapy app in the mental health sector. Only 16.2% of practitioners had any previous experience with blended therapy, showing that this concept is not widely used in Austria. Even though there was very little internet anxiety in our samples, both the general population and practitioners seem to be cautious, but slightly positive about blended therapy. An earlier review about studies using the UTAUT found an average acceptance of low to moderate (M = 2.82, SD = 1.12, scale range 1–5). This review was conducted in 2021, analyzing studies between 2014 and 2020. Our results show that both practitioners and general population had a higher acceptance rate (3.36 and 3.75, respectively), suggesting that blended therapy has become more accepted nowadays than 5–10 years ago. This might be due to several reasons, such as the need of technology during the COVID-19 pandemic or the ever-increasing use of technology. Yet, it needs to be mentioned that comparisons might be limited due to diverse settings and outcomes of the studies. Practitioners are most worried about the therapeutic process and their work-life balance, while the general population worries that they would be too overwhelmed by the concept, mainly due to the time investment. Tracking, recording and reminding functions (e.g., for mood, homework) are seen as especially valuable features and accessibility was deemed the biggest advantage by both samples. Interestingly, in our open format, our sample of general population and practitioners often suggested the same additional things: a diary function, encouraging messages, relaxation techniques, reminder and tracking functions (e.g., medication, exercising) as well as literature recommendations were suggested for interesting features, while insufficient technical equipment and fear of mandatory use was mentioned as concerns. An example regarding exercise tracking/reminding can be found in a RCT that found that in-person counselling and the use of a mobile app significantly increased physical activity for the experimental group compared to a control group [29]. Yet, the study showed only short-term effects, with no significant difference after 3 months, highlighting the importance of testing long-term effects.

Even though it is not yet clear that blended therapy is superior to face-to-face therapy, it might have advantages for practitioners [15] and might help patients to internalize therapeutic content between sessions with a practitioner. As one focus group participant in a qualitative study by Richards et al. [25] said about the impact of technology in a therapeutic context: “the voice of therapy is going to stay with the client and […] they will become their own therapist”.

The literature supports the notion that practitioners do not use apps for several reasons, such as too little information or no knowledge about existing apps [24], yet evaluations about apps already being used show that they are largely accepted and liked [e.g. 30]. Our results suggest that blended therapy is not common in Austria and that this might be connected to practitioners’ cautiousness towards this form of therapy. Yet, research shows that positive attitudes about ehealth and blended therapy from practitioners are positively linked to patients’ attitudes [31]. Patients are more likely to use web-based therapy recommended by practitioners [32], which influences whether and how much patients use an mhealth app [22]. All in all, practitioners play a major role in the uptake and adherence to such programs.

Practically, several directions might help with changing the attitude of users of blended therapy. Firstly, the literature on blended therapy is largely supportive [17, 18]. For example, blended approaches can lead to superior treatment outcomes according to Lindhiem et al. [33], who conducted a review of 26 empirical studies. A more positive awareness could be sought especially in practitioners through advocating these findings, as more awareness for these positive aspects might help with diminishing the concerns of Austrian practitioners. Our results show that practitioners with experience with blended therapy had a higher acceptance, supporting the notion that experience and knowledge could lead to higher acceptance. Secondly, given the concerns about payments – the general public is concerned about additional cost and practitioners are concerned about problems with the billability of blended services – it is important that blended therapy apps are embedded in health insurance policies. One example close at hand are digital health applications (*Digitale Gesundheitsanwendungen*, DiGA), which already exist in Germany but not in Austria. With DIGAs, a blended therapy app could be prescribed by a doctor via the health insurance, eliminating costs for patients as well as practitioners. This official process might also eliminate some fears about data security and time management, which were some of the biggest concerns for both the general population and practitioners.

### Limitation and future research

While this study gives a clearer insight into attitudes about blended therapy in Austria, some limitations can be acknowledged. Our sample was a convenience sample, meaning for example that we could not fully cover opinions from all occupations of practitioners. Due to the convenience sample, there was a predominance of women in the practitioner sample. Furthermore, most of our sample of practitioners work with children and adolescents. Arguably, this is an area where blended therapy might be especially important, as younger generations become more and more inclined towards technology and might benefit from a blended approach. As our general sample consisted of adults, examining the attitudes of adolescents and children and their legal guardians towards blended therapy might be a topic for future research. Furthermore, to increase uptake and to gather more evidence, blended therapy concepts should be developed and tested in an Austrian context.

## Conclusion

This study showed that blended therapy in Austria is not used widely and, while cautiously positive, both practitioners and the general population have some concerns and fears about such a form of therapy. Yet, our sample had various ideas what a blended therapy app could include that would help with traditional face-to-face therapy, which could steer blended therapy development in Austria.

## Supplementary Information

Questionnaires
