# Supplementary material for: Attitudes of the general population and mental health practitioners towards blended therapy in Austria
Source: Wien Klin Wochenschr. 2024 Jul 22;137(3-4):118–25. doi: 10.1007/s00508-024-02391-9 (PMC11794416; doi:10.1007/s00508-024-02391-9)
Supplement: Supplementary file 1 — Questionnaires [file 508_2024_2391_MOESM1_ESM.docx]

| **Practitioners** | |
| --- | --- |
| **German** | **English translation** |
| Ich möchte an der Studie teilnehmen. | I want to participate in the study. |
| Geschlecht:  **weiblich**  **männlich**  **non-binär** | Gender:  **Female**  **Male**  **Diverse** |
| Alter: | Age: |
| Ich bin...  **Ärzt:in**  **Psychotherapeut:in**  **Klinische(r) Psycholog:in**  **Ergotherapeut:in**  **Sozialarbeiter*in**  **In Ausbildung:**  **Anderes:** | I am...  **Doctor**  **Psychotherapist**  **Clinical psychologist**  **Occupational therapist**  **Social worker**  **In training:**  **Other:** |
| Ich arbeite in:  **Wien**  **Niederösterreich**  **Burgenland**  **Voralberg**  **Oberösterreich**  **Salzburg**  **Tirol**  **Steiermark**  **Kärnten**  **Anderes** | I work in:  **Vienna**  **Lower Austria**  **Burgenland**  **Voralberg**  **Upper Austria**  **Salzburg**  **Tyrol**  **Styria**  **Carinthia**  **Other** |
| Meine Patient:innen sind hauptsächlich: (Mehrfachantwort möglich)  **Kinder**  **Jugendliche**  **Junge Erwachsene**  **Ältere Personen** | My patients are mainly: (multiple answers possible)  **Children**  **Adolescents**  **Young adults**  **Older people** |
| Ich arbeite in einer...  **Klinik**  **Privatordination**  **Kassenordination**  **Soziale Einrichtung (z.B. Caritas)**  **Anderes:** | I work in a...  **Clinic**  **Private practice**  **Health insurance practice**  **Social organisation (e.g. Caritas)**  **Other:** |
| Haben Sie schon mit Blended Therapy gearbeitet oder arbeiten Sie zur Zeit mit Blended Therapy?  **ja**  **nein** | Have you already worked with blended therapy or are you currently working with blended therapy?  **Yes**  **No** |
| Blended Therapy ist eine Kombination von Offline- und Online-Elementen in einem Behandlungsprozess, beinhaltet also klassische face-to-face Therapie mit Online Interventionen. Ein Beispiel wäre eine Smartphone-App, die während dem Behandlungszeitraum zusätzlich genutzt wird.  Welche Inhalte fänden Sie in einer App bei Blended Therapy vorteilhaft?  **Monitoring der Stimmung**  **Vermerken der „Hausaufgaben“**  **Vermerken der therapeutischen Inhalte**  **Gewohnheits-Tracker (z.B. Sport / Mahlzeiten)**  **Funktion zum Ziele formulieren / tracken**  **Notfallnummern**  **Chat / Videocall Funktion (mit Ihnen)**  **Chat / Forum Funktion (für alle Patient:innen)** | Blended therapy is a combination of offline and online elements in a treatment process, i.e. it includes classic face-to-face therapy with online interventions. One example would be a smartphone app that is also used during the treatment period.  What content would you find beneficial in an app for blended therapy?  **Monitoring the mood**  **Recording the ‘homework’**  **Recording the therapeutic content**  **Habit tracker (e.g. sport / meals)**  **Function for formulating / tracking goals**  **Emergency numbers**  **Chat / video call function (with you)**  **Chat / forum function (for all patients)** |
| Haben Sie sonst noch Ideen / Verschläge / Wünsche, welche Funktionen in einer App sein könnten? | Do you have any other ideas / suggestions / wishes as to which functions could be included in an app? |
| Im Folgenden geht es um Ihre Einstellungen zur Blended Therapy.   Blended Therapy ist eine Kombination von Offline- und Online-Elementen in einem Behandlungsprozess, beinhaltet also klassische face-to-face Therapie mit Online Interventionen. Ein Beispiel wäre eine Smartphone-App, die während dem Behandlungszeitraum zusätzlich genutzt wird.  [trifft überhaupt nicht zu; trifft eher nicht zu; teils/teils; trifft eher zu; trifft komplett zu]  **Blended Therapy könnte die Wirksamkeit meiner Behandlungen verbessern.**  **Blended Therapy könnte meine Arbeit unterstützen und meine Produktivität steigern.**  **Blended Therapy könnte meinen Patient:innen allgemein helfen.**  **Blended Therapy würde die therapeutische Beziehung beeinträchtigen.**  **Die Bedürfnisse der Patient:innen könnten mit Blended Therapy nicht ausreichend berücksichtigt werden.**  **Meine Möglichkeiten, in bestimmten Situationen zu reagieren, wären mit Blended Therapy eingeschränkt.**  **Blended Therapy wäre für meine Arbeit nicht förderlich, weil deren Entwicklung nicht praxisorientiert ist.**  **Ich kann mir nicht vorstellen, Blended Therapy einzusetzen, weil es eine Gefahr für die therapeutische Arbeit darstellen würde.** | The following is about your attitudes towards blended therapy.  Blended therapy is a combination of offline and online elements in a treatment process, i.e. it includes classic face-to-face therapy with online interventions. An example would be a smartphone app that is also used during the treatment period.  [strongly disagree; somewhat disagree; partly/partly agree; somewhat agree; completely agree]  **Performance expectancy**  **Blended therapy would improve the effectiveness of my treatments.**  **Blended therapy could support my work and increase my productivity.**  **Blended therapy would help my patients generally.**  **I expect blended therapy would hinder the therapeutic relationship’.**  **Patients’ needs cannot be sufficiently targeted by blended therapy’.**  **My possibilities to react in certain situations are restricted in blended therapy’.**  **Blended therapy will not be beneficial for my work, because its development is not practice orientated’.**  **I cannot imagine to use blended therapy, because of its danger for the therapeutic work’** |
| Im Folgenden geht es um Ihre Einstellungen zur Blended Therapy.   Blended Therapy ist eine Kombination von Offline- und Online-Elementen in einem Behandlungsprozess, beinhaltet also klassische face-to-face Therapie mit Online Interventionen. Ein Beispiel wäre eine Smartphone-App, die während dem Behandlungszeitraum zusätzlich genutzt wird.  [trifft überhaupt nicht zu; trifft eher nicht zu; teils/teils; trifft eher zu; trifft komplett zu]  **Ich glaube, die Anwendung von Blended Therapy wäre einfach.**  **Ich glaube, ich könnte mit Blended Therapy leicht umgehen.**  **Ich glaube, die Anwendung von Blended Therapy wäre einfach und verständlich.**  **Ich glaube, es wäre schwierig, die Compliance der Patient:innen zu erreichen.**  **Ich glaube, die Anwendung von Blended Therapy würde eine höhere Arbeitsbelastung für mich bedeuten.**  **Ich glaube, es wäre schwierig, Blended Therapy in meine Arbeit zu integrieren.** | The following is about your attitudes towards blended therapy.  Blended therapy is a combination of offline and online elements in a treatment process, i.e. it includes classic face-to-face therapy with online interventions. An example would be a smartphone app that is also used during the treatment period.  [strongly disagree; somewhat disagree; partly/partly agree; somewhat agree; completely agree]  Effort expectancy  **Use of blended therapy would be simple.**  **I could handle blended therapy easily.**  **Use of blended therapy would be easy and comprehensible.**  **Creating patients’ compliance would be difficult’.**  **Use of blended therapy would create a higher workload for myself’**  **It would be hard to integrate blended therapy in my work’.** |
| Im Folgenden geht es um Ihre Einstellungen zur Blended Therapy.   Blended Therapy ist eine Kombination von Offline- und Online-Elementen in einem Behandlungsprozess, beinhaltet also klassische face-to-face Therapie mit Online Interventionen. Ein Beispiel wäre eine Smartphone-App, die während dem Behandlungszeitraum zusätzlich genutzt wird.  [trifft überhaupt nicht zu; trifft eher nicht zu; teils/teils; trifft eher zu; trifft komplett zu]  **Meine Kolleg:innen würden mir zu Blended Therapy raten.**  **Vorgesetzte oder erfahrenen Kolleg:innen würden mir raten, Blended Therapy zu nutzen.**  **Meine Kolleg:innen würden mir von der Anwendung von Blended Therapy abraten.** | The following is about your attitudes towards blended therapy.  Blended therapy is a combination of offline and online elements in a treatment process, i.e. it includes classic face-to-face therapy with online interventions. An example would be a smartphone app that is also used during the treatment period.  [strongly disagree; somewhat disagree; partly/partly agree; somewhat agree; completely agree]  Social influence  **My colleagues would advise me to use blended therapy.**  **My supervisor or experienced colleagues would advise me to use blended therapy.**  **My colleagues would discourage me from using blended therapy’.** |
| Im Folgenden geht es um Ihre Einstellungen zur Blended Therapy.   Blended Therapy ist eine Kombination von Offline- und Online-Elementen in einem Behandlungsprozess, beinhaltet also klassische face-to-face Therapie mit Online Interventionen. Ein Beispiel wäre eine Smartphone-App, die während dem Behandlungszeitraum zusätzlich genutzt wird.  [trifft überhaupt nicht zu; trifft eher nicht zu; teils/teils; trifft eher zu; trifft komplett zu]  **Ich denke, ich würde Unterstützung erhalten, wenn ich auf technische Probleme stoße.**  **Ich glaube, ich bin technisch versiert genug für den Einsatz von Blended Therapy.**  **Ich glaube, Blended Therapy kann Probleme mit der Sicherheit von Daten und Privatsphäre verursachen.**  **Ich erwarte zusätzliche Kosten, wenn ich Blended Therapy einsetzen würde.**  **Ich erwarte zusätzliche Kosten für meine Patient:innen, wenn ich Blended Therapy einsetzen würde.**  **Die Handhabung der Blended Therapy wäre für meine Patient:innen schwierig.**  **Meine Patient:innen sind nicht technisch versiert genug, um Blended Therapy zu nutzen.** | The following is about your attitudes towards blended therapy.  Blended therapy is a combination of offline and online elements in a treatment process, i.e. it includes classic face-to-face therapy with online interventions. An example would be a smartphone app that is also used during the treatment period.  [strongly disagree; somewhat disagree; partly/partly agree; somewhat agree; completely agree]  Facilitating conditions  **I would get support, if I encounter technical problems.**  **I fulfill all technical requirements to use blended therapy.**  **Blended therapy can cause problems with data and privacy security’.**  **I expect additional costs, if I use blended therapy.**  **I expect additional costs for my patients, if I use blended therapy´.**  **Handling of blended therapy would be difficult for my patients’.**  **My patients do not fulfill the technical requirements to use blended therapy’.** |
| Im Folgenden geht es um Ihre Einstellungen zur Blended Therapy.   Blended Therapy ist eine Kombination von Offline- und Online-Elementen in einem Behandlungsprozess, beinhaltet also klassische face-to-face Therapie mit Online Interventionen. Ein Beispiel wäre eine Smartphone-App, die während dem Behandlungszeitraum zusätzlich genutzt wird.  [trifft überhaupt nicht zu; trifft eher nicht zu; teils/teils; trifft eher zu; trifft komplett zu]  **Das Internet hat etwas Bedrohliches für mich.**  **Ich habe Angst, bei der Nutzung des Internets einen unwiderruflichen Fehler zu machen.**  **Ich bin sehr besorgt, wenn ich das Internet benutze.** | The following is about your attitudes towards blended therapy.  Blended therapy is a combination of offline and online elements in a treatment process, i.e. it includes classic face-to-face therapy with online interventions. An example would be a smartphone app that is also used during the treatment period.  [strongly disagree; somewhat disagree; partly/partly agree; somewhat agree; completely agree]  Internet anxiety  **The internet has something threatening to me.**  **I am afraid making an irrevocable mistake while using the internet.**  **I am very concerned when I use the internet.** |
| Wie sehr empfinden Sie dien achfolgenden Punkte als VORTEILE bei der Nutzung von BlendedTherapy? (Mehrfachauswahl möglich) [sehr; ein bisschen; teils/teils; nicht so sehr; gar nicht]  **Erreichbarkeit (z.B. für ländliche Gebiete)**  **Vertiefung des therapeutischen Prozesses**  **Vertiefung der therapeutischen Beziehung**  **Monitoring der Patient:innen (z.B. Stimmung)**  **Kontinuierliche Beteiligung der Patient:innen zwischen den Einheiten (z.B. „Hausaufgaben“)** | How much do you consider the following points to be ADVANTAGES when using Blended Therapy? (multiple choice possible) [very much; a little; partly/partly; not so much; not at all]  **Accessibility (e.g. for rural areas)**  **Deepening of the therapeutic process**  **Deepening of the therapeutic relationship**  **Monitoring of the patient (e.g. mood)**  **Continuous involvement of patients between sessions (e.g. ‘homework’)** |
| Wie sehr empfinden Sie die nachfolgenden Punkte als HINDERNIS zur Nutzung von Blended Therapy? (Mehrfachauswahl möglich) [sehr; ein bisschen; teils/teils; nicht so sehr; gar nicht]  **Datensicherheit**  **Zeitaufwand**  **Therapeutischer Prozess**  **Therapeutische Beziehung**  **Depersonalisierung**  **Work-Life Balance**  **Überforderung der Patient:innen**  **Abrechenbarkeit (finanzielle Hindernisse)**  **Weitere Hindernisse:** | How much do you consider the following points to be OBSTACLES to the use of blended therapy? (multiple choice possible) [very much; a little; partly/partly; not so much; not at all]  **Data security**  **Time required**  **Therapeutic process**  **Therapeutic relationship**  **Depersonalisation**  **Work-life balance**  **Excessive demands on the patient**  **Billability (financial obstacles)**  **Further obstacles:** |

| **General Population** | |
| --- | --- |
| **German** | **English translation** |
| Ich möchte an der Studie teilnehmen. | I want to participate in the study. |
| Geschlecht:  **weiblich**  **männlich**  **divers** | Gender:  **Female**  **Male**  **Diverse** |
| Alter: | Age: |
| Ich lebe in:  **Wien**  **Niederösterreich**  **Burgenland**  **Voralberg**  **Oberösterreich**  **Salzburg**  **Tirol**  **Steiermark**  **Kärnten**  **Anderes** | I live in:  **Vienna**  **Lower Austria**  **Burgenland**  **Voralberg**  **Upper Austria**  **Salzburg**  **Tyrol**  **Styria**  **Carinthia**  **Other** |
| Befinden Sie sich derzeit in psychologischer therapeutischer Behandlung?  **ja**  **nein** | Are you currently undergoing psychological therapy?  **Yes**  **No** |
| Haben Sie eine diagnostizierte psychische Erkrankung?  **nein**  **ja, Depression**  **ja, Angststörung**  **ja, anderes:** | Do you have a diagnosed mental illness?  **No**  **Yes, depression**  **Yes, anxiety disorder**  **Yes, other:** |
| Blended Therapy ist eine Kombination von Offline- und Online-Elementen in einem Behandlungsprozess, beinhaltet also klassische face-to-face Therapie mit Online Interventionen. Ein Beispiel wäre eine Smartphone-App, die während dem Behandlungszeitraum zusätzlich genutzt wird.  Welche Inhalte fänden Sie in einer App bei Blended Therapy vorteilhaft?  **Monitoring der Stimmung**  **Vermerken der „Hausaufgaben“**  **Vermerken der therapeutischen Inhalte**  **Gewohnheits-Tracker (z.B. Sport / Mahlzeiten)**  **Funktion zum Ziele formulieren / tracken**  **Notfallnummern**  **Chat / Videocall Funktion (mit Ihnen)**  **Chat / Forum Funktion (für alle Patient:innen)** | Blended therapy is a combination of offline and online elements in a treatment process, i.e. it includes classic face-to-face therapy with online interventions. One example would be a smartphone app that is also used during the treatment period.  What content would you find beneficial in an app for blended therapy?  **Monitoring the mood**  **Recording the ‘homework’**  **Recording the therapeutic content**  **Habit tracker (e.g. sport / meals)**  **Function for formulating / tracking goals**  **Emergency numbers**  **Chat / video call function (with you)**  **Chat / forum function (for all patients)** |
| Haben Sie sonst noch Ideen / Verschläge / Wünsche, welche Funktionen ineiner App sein könnten? | Do you have any other ideas / suggestions / wishes as to which functions could be included in an app? |
| Im Folgenden geht es um Ihre Einstellungen zur Blended Therapy.   Blended Therapy ist eine Kombination von Offline- und Online-Elementen in einem Behandlungsprozess, beinhaltet also klassische face-to-face Therapie mit Online Interventionen. Ein Beispiel wäre eine Smartphone-App, die während dem Behandlungszeitraum zusätzlich genutzt wird.  [trifft überhaupt nicht zu; trifft eher nicht zu; teils/teils; trifft eher zu; trifft komplett zu]  **Blended Therapy könnte die Wirksamkeit meiner Behandlung verbessern.**  **Blended Therapy könnte mich bei der Erreichung von Zielen im Alltag unterstützen.**  **Blended Therapy könnte mir allgemein helfen.**  **Blended Therapy würde die Beziehung zu meiner/m Therapeut:in beeinträchtigen.**  **Meine Bedürfnisse könnten mit Blended Therapy nicht ausreichend berücksichtigt werden.**  **Die Möglichkeiten meiner/s Therapeut:in, in bestimmten Situationen zu reagieren, wären mit Blended Therapy eingeschränkt.**  **Blended Therapy wäre für mich nicht förderlich, weil deren Entwicklung nicht praxisorientiert ist.**  **Ich kann mir nicht vorstellen, dass eine Therapie, die Online Interventionen beinhaltet, funktionieren würde.** | The following is about your attitudes towards blended therapy.  Blended therapy is a combination of offline and online elements in a treatment process, i.e. it includes classic face-to-face therapy with online interventions. An example would be a smartphone app that is also used during the treatment period.  [strongly disagree; somewhat disagree; partly/somewhat agree; somewhat agree; completely agree]  Performance expectancy  **Blended therapy could improve the effectiveness of my treatment.**  **Blended therapy could support me in achieving goals in everyday life.**  **Blended therapy could help me in general.**  **Blended therapy would affect my relationship with my therapist.**  **My needs could not be adequately addressed with blended therapy.**  **My therapist's ability to react in certain situations would be limited with blended therapy.**  **Blended therapy would not be beneficial for me because its development is not practice-orientated.**  **I can't imagine that a therapy that includes online interventions would work.** |
| Im Folgenden geht es um Ihre Einstellungen zur Blended Therapy.   Blended Therapy ist eine Kombination von Offline- und Online-Elementen in einem Behandlungsprozess, beinhaltet also klassische face-to-face Therapie mit Online Interventionen. Ein Beispiel wäre eine Smartphone-App, die während dem Behandlungszeitraum zusätzlich genutzt wird.  [trifft überhaupt nicht zu; trifft eher nicht zu; teils/teils; trifft eher zu; trifft komplett zu]  **Ich glaube, die Anwendung von Blended Therapy wäre einfach.**  **Ich glaube, ich könnte mit Blended Therapy leicht umgehen.**  **Ich glaube, die Anwendung von Blended Therapy wäre einfach und verständlich.**  **Ich glaube, die Anwendung von Blended Therapy würde mich im Alltag stören.**  **Ich glaube, es wäre schwierig, Blended Therapy in meine Therapie zu integrieren.** | The following is about your attitudes towards blended therapy.  Blended therapy is a combination of offline and online elements in a treatment process, i.e. it includes classic face-to-face therapy with online interventions. An example would be a smartphone app that is also used during the treatment period.  [strongly disagree; somewhat disagree; partly/somewhat agree; somewhat agree; completely agree]  Effort expectancy  **I think it would be easy to use blended therapy.**  **I think I could handle blended therapy easily.**  **I think the application of blended therapy would be simple and understandable.**  **I think using Blended Therapy would interfere with my everyday life.**  **I think it would be difficult to integrate Blended Therapy into my therapy.** |
| Im Folgenden geht es um Ihre Einstellungen zur Blended Therapy.   Blended Therapy ist eine Kombination von Offline- und Online-Elementen in einem Behandlungsprozess, beinhaltet also klassische face-to-face Therapie mit Online Interventionen. Ein Beispiel wäre eine Smartphone-App, die während dem Behandlungszeitraum zusätzlich genutzt wird.  [trifft überhaupt nicht zu; trifft eher nicht zu; teils/teils; trifft eher zu; trifft komplett zu]  **Ich denke, ich würde Unterstützung erhalten, wenn ich auf technische Probleme stoße.**  **Ich glaube, ich bin technisch versiert genug für den Einsatz von Blended Therapy.**  **Ich glaube, Blended Therapy kann Probleme mit der Sicherheit von Daten und Privatsphäre verursachen.**  **Ich erwarte zusätzliche Kosten, wenn ich Blended Therapy einsetzen würde.**  **Die Handhabung der Blended Therapy wäre schwierig.**  **Mein(e) Therapeut:in ist nicht technisch versiert genug, um Blended Therapy zu nutzen.** | The following is about your attitudes towards blended therapy.  Blended therapy is a combination of offline and online elements in a treatment process, i.e. it includes classic face-to-face therapy with online interventions. An example would be a smartphone app that is also used during the treatment period.  [strongly disagree; somewhat disagree; partly/somewhat agree; somewhat agree; completely agree]  Facilitating conditions  **I think I would receive support if I encountered technical problems.**  **I think I am technically skilled enough to use blended therapy.**  **I think blended therapy can cause problems with data security and privacy.**  **I anticipate additional costs if I were to use blended therapy.**  **Blended therapy would be difficult to manage.**  **My therapist is not tech-savvy enough to use blended therapy.** |
| Im Folgenden geht es um Ihre Einstellungen zur Blended Therapy.   Blended Therapy ist eine Kombination von Offline- und Online-Elementen in einem Behandlungsprozess, beinhaltet also klassische face-to-face Therapie mit Online Interventionen. Ein Beispiel wäre eine Smartphone-App, die während dem Behandlungszeitraum zusätzlich genutzt wird.  [trifft überhaupt nicht zu; trifft eher nicht zu; teils/teils; trifft eher zu; trifft komplett zu]  **Das Internet hat etwas Bedrohliches für mich.**  **Ich habe Angst, bei der Nutzung des Internets einen unwiderruflichen Fehler zu machen.**  **Ich bin sehr besorgt, wenn ich das Internet benutze.** | The following is about your attitudes towards blended therapy.  Blended therapy is a combination of offline and online elements in a treatment process, i.e. it includes classic face-to-face therapy with online interventions. An example would be a smartphone app that is also used during the treatment period.  [strongly disagree; somewhat disagree; partly/somewhat agree; somewhat agree; completely agree]  Internet anxiety  **There is something threatening about the internet for me.**  **I am afraid of making an irrevocable mistake when using the Internet.**  **I am very worried when I use the Internet.** |
| Wie sehr empfinden Sie die nachfolgenden Punkte als VORTEILE bei der Nutzung von Blended Therapy? (Mehrfachauswahl möglich) [sehr; ein bisschen; teils/teils; nicht so sehr; gar nicht]  **Erreichbarkeit (z.B. für ländliche Gebiete)**  **Vertiefung Inhalte der Offline Sitzungen**  **Vertiefung der Beziehung zu meiner/m Therapeut:in**  **Tracking (z.b. Stimmung, Sportverhalten)**  **Monitoring von „Hausaufgaben“ aus den Therapiesitzungen**  **Erinnerungen für Medikamente, Termine, etc.**  **Edukative Videos**  **Weitere Vorteile:** | How much do you consider the following points to be ADVANTAGES when using blended therapy? (Multiple choice possible) [very much; a little; partly/partly; not so much; not at all]  **Accessibility (e.g. for rural areas)**  **Deepening the content of offline sessions**  **Deepening the relationship with my therapist**  **Tracking (e.g. mood, sports behaviour)**  **Monitoring of ‘homework’ from the therapy sessions**  **Reminders for medication, appointments, etc.**  **Educational videos**  **Further advantages:** |
| Wie sehr empfinden Sie die nachfolgenden Punkte als HINDERNIS zur Nutzung von Blended Therapy? (Mehrfachauswahl möglich) [sehr; ein bisschen; teils/teils; nicht so sehr; gar nicht]  **Datensicherheit**  **Zeitaufwand**  **Geld**  **Beziehung zu meiner/m Therapeut:in**  **Online Kontakt nicht so gut wie Offline Kontakt**  **Überforderung**  **Weitere Hindernisse:** | How much do you consider the following points to be OBSTACLES to the use of blended therapy? (multiple choice possible) [very much; a little; partly/partly; not so much; not at all]  **Data security**  **Time expenditure**  **Money**  **Relationship with my therapist**  **Online contact not as good as offline contact**  **Excessive demands**  **Other obstacles:** |
